# The Cognitive Building Blocks of Emotion Regulation: Ability to Update Working Memory Moderates the Efficacy of Rumination and Reappraisal on Emotion

**DOI:** 10.1371/journal.pone.0069071

**Published:** 2013-07-18

**Authors:** Madeline Lee Pe, Filip Raes, Peter Kuppens

**Affiliations:** 1 Faculty of Psychology and Educational Sciences, KU Leuven, Leuven, Belgium; 2 School of Psychological Science, University of Melbourne, Parkville, Australia; University of Bologna, Italy

## Abstract

The ability to regulate emotions is a critical component of healthy emotional functioning. Therefore, it is important to determine factors that contribute to the efficacy of emotion regulation. The present article examined whether the ability to update emotional information in working memory is a predictor of the efficacy of rumination and reappraisal on affective experience both at the trait level (Study 1) and in daily life (Study 2). In both studies, results revealed that the relationship between use of reappraisal and high arousal negative emotions was moderated by updating ability. Specifically, use of reappraisal was associated with decreased high arousal negative emotions for participants with high updating ability, while no significant relationship was found for those with low updating ability. In addition, both studies also revealed that the relationship between rumination and high arousal negative emotions was moderated by updating ability. In general, use of rumination was associated with elevated high arousal negative emotions. However, this relationship was blunted for participants with high updating ability. That is, use of rumination was associated with less elevated high arousal negative emotions for participants with high updating ability. These results identify the ability to update emotional information in working memory as a crucial process modulating the efficacy of emotion regulation efforts.

## Introduction

The ability to regulate emotions plays a key role in determining healthy from problematic emotional functioning. Although the past decade has seen an upsurge of research into the use and consequences of emotion regulation, much less is known about the factors that make people regulate their emotions effectively. In other words, what determines the outcome of regulating one’s emotions?

One promising avenue lies in executive functions (see also [1]). Indeed, executive functions or cognitive control are considered to play a central role in emotion regulation [1]–[3]. There is a growing number of empirical studies showing that individual differences in cognitive control are related to the use of various emotion regulation strategies. For instance, the ability to control negative information in working memory (WM) has been associated with the habitual use of both rumination (e.g., [4]–[5]) and reappraisal (e.g., [6]).

Although studies like these give invaluable insight into the cognitive underpinnings underlying the use of specific emotion regulation strategies, it is at least equally important to understand whether these cognitive abilities determine the efficacy of emotion regulation strategies for changing one’s emotions [1]. That is, in addition to examining whether deficits in controlling negative material in WM would increase or decrease the use of certain emotion regulation strategies (e.g., rumination), it should also be examined to what extent and how such deficits modulate the effect of such emotion regulation strategies on emotional experience [7]. From this perspective, emotion regulation is viewed as a higher cognitive activity that necessitates the support of basic cognitive processes to determine its efficacy.

In the present article, two studies are presented that addressed whether individual differences in controlling emotional information in WM indeed relate to the efficacy of emotion regulation strategies on emotional experience. More precisely, using trait questionnaires (Study 1) and experience sampling methodology (Study 2), we examined whether the ability to update emotional information in WM moderates the efficacy of rumination and reappraisal on the experience of positive and negative emotions.

### Updating and Emotion Regulation

In trying to understand the role of cognitive processes in emotion regulation, we focus on (depressive) rumination and reappraisal. Whereas (depressive) rumination is defined as repetitively thinking about negative feelings, their possible causes, meanings and consequences [8], reappraisal is defined as viewing emotional events from a different perspective so as to lessen their emotional impact [9]. Both strategies are implemented cognitively and involve sustained attention on emotional experience [10], making them prime candidates to be affected by the cognitive processes discussed above. Yet, they are also clearly distinct. Habitual use of rumination is related to enhanced negative thinking, impaired problem solving, exacerbated and prolonged distress, and makes one more vulnerable to the development of psychopathology (e.g., depression) [8], while habitual use of reappraisal is generally associated with more desirable outcomes on average such as reduced negative emotions, increased positive emotions, better interpersonal functioning and well-being [9]. As such, the focus on these two strategies allows testing of predictions on the role of updating on both adaptive and maladaptive forms of emotion regulation on emotional experience.

Here, we argue that the efficacy of such emotion regulation actions critically relies on the extent to which the contents in WM are able to change to accommodate new information as it becomes available. For instance, when a negative event is experienced (e.g., failing an exam), negative thoughts are activated in WM (e.g., I’m not good enough), and negative emotions are experienced (e.g., sad). In order to reduce the experience of negative emotions, there is a need to modify or update these negative thoughts with newer information that is either more neutral or positive (e.g., I failed, but that does not determine my success or failure as a student). Updating, which refers to the ability to change the contents of WM to accommodate new information [11], is the executive function that is involved in such cognitive activity.

### Updating and Reappraisal

The importance of being able to update information in WM is crucial to effective reappraisal. Since reappraisal involves *changing* one’s current perspective of an emotion-eliciting event, updating is necessary in order to form another perspective by replacing the current perspective with a newer and more relevant one. In other words, updating is necessary in order to effectively reappraise.

To our knowledge, only one study has tested this hypothesis. In a study by Schmeichel, Volokhov and Demaree [12], they showed that the efficacy of reappraisal was moderated by updating (as measured by a non-emotional n-back task). That is, people with high (vs. low) updating ability were more successful at reappraising a disgusting film in the lab as non-emotional, as evidenced by their experience and expression of less disgust in response to the film.

We therefore hypothesize that updating will moderate the relationship between reappraisal and emotional experience, such that people with a high (vs. low) updating ability will experience less negative and more positive emotions when reappraising.

### Updating and Rumination

(Depressive) rumination involves the process of thinking *repetitively* about one’s feelings and problems [8]. This suggests that when ruminating, people continuously dwell on (negative) information that is already present in WM [13], resulting in a heightened experience of negative affect [14]–[15].

We propose that updating is one executive process that could help break the ruminative process by reducing the elaboration of negative material in WM [13]. Indeed, since rumination is about recycling thoughts in WM, then having the ability to accommodate new information in WM (through updating) would allow newer and possibly mood-incongruent thoughts to feed into the ruminative process. This would aid in minimizing the maladaptive consequences of rumination (i.e., experiencing less elevated negative and less decreased positive emotions). Conversely, the inability to update information in WM would lead to rumination having a continuous detrimental impact on affective experience.

Whereas there is evidence that updating and rumination are related [16], no study has examined whether updating also modulates the relationship between rumination and emotional experience. We hypothesize that updating will moderate the relationship between rumination and emotional experience, such that people with a high (vs. low) updating ability will experience less elevated negative and less decreased positive emotions when ruminating.

### The Present Study

As far as we know, the study done by Schmeichel and colleagues [12] using the *n*-back is the only empirical evidence so far that identifies updating as a moderator of the efficacy of reappraisal. Therefore, as a first goal, we aimed to provide empirical replication for the role of updating on the efficacy of emotion regulation by using an emotional variant of the *n*-back as a measure of updating. The underlying rationale is that a task that specifically involves valenced emotional information in working memory would be particularly relevant for picking up the processes operating during emotion regulation. Second, the relationship between updating and efficacy of reappraisal has only been examined using laboratory studies [12]. While it is definitely important to study people’s emotional responses to standardized stimuli in the lab, it is nevertheless crucial to replicate lab findings in the context of the complexities encountered in daily life [17]. After all, it is there that psychological phenomena eventually play out and affect people’s well-being. Lastly, we wanted to examine whether the moderating effect of updating ability would extend to the case rumination. That is, if updating is indeed an important determinant of effective reappraisal, we project that this would also aid in curbing or attenuating the deleterious effect of rumination on emotional experience.

In sum, with the present research, we sought to examine whether updating of emotional information in WM (as measured by an emotional *n*-back task [18]) would be associated with the efficacy of rumination and reappraisal on emotional experience both at the trait level (Study 1) and in daily life (Study 2). We hypothesized that updating of emotional information in WM would moderate the efficacy of both reappraisal and rumination on emotional experience. That is, we expected that people who are better at updating emotional information would experience more decreased negative affect and more elevated positive affect when reappraising; and less elevated negative affect and less decreased positive affect when ruminating.

## Study 1: Trait Level

Study 1 tested the hypothesis that at the trait level, updating of emotional information would moderate the relationship between reappraisal and rumination, on the one hand, and positive and negative emotions, on the other hand. Participants completed an emotional *n*-back task and responded to questionnaires regarding their feelings during the past week, and their habitual use of reappraisal and rumination, among others.

### Ethics Statement

The study was conducted in December 2011 as part of a large collective research program organized and approved by the Faculty of Psychology and Educational Sciences, KU Leuven, Belgium. All first year psychology students are invited to participate in this research in exchange for course credits. Written informed consent was obtained from all participants at the start of the program.

In accordance with the “Law of 7 May 2004 concerning experiments on the human person,” (https://ppw.kuleuven.be/intern/ethischecommissie/wet) an authorization is necessary from the Medical Ethics Committee of the University Hospitals Leuven, Faculty of Medicine for experiments that “touches the person in their essence.” These experiments mean studies which include physical changes such as the need to breathe faster, painful stimuli, using deception and other (taken from https://ppw.kuleuven.be/intern/ethischecommissie/index and translated to English).

The present study used questionnaires and a computer task that fall within these conditions, and therefore did not necessitate approval from the ethics committee. However, as of June 2012, the University has decided that all studies within the collective research program would require ethical approval. Since then, we have applied for and have been granted ethical approval for studies using similar participants and the same computer task and questionnaires.

### Participants

Two hundred twenty-five first year psychology students participated in this study. Four participants did not have complete data and were eliminated from the study, leaving a sample of 221 (184 women; *M_age_*  = 18.47, *SD_age_*  = 1.22). Participants earned partial course credit for their participation in the study.

### Procedure

The study was administered in groups of 25, with each session lasting for a maximum of 30 minutes. All participants were first required to respond to the emotional *n-*back task, which was administered on a desktop computer. After completing the computer task, they responded to trait questionnaires using paper and pencil.

### Measures

#### Emotional n-back

Participants completed the emotional *n*-back task as a measure of updating emotional information [18]. This task was adapted from Levens and Gotlib [13], but words instead of faces were used as stimuli. A total of 47 positive and 49 negative words were selected from the Affective Norms of English Words list [19] and translated into Dutch. Words were identified as negative and positive if their valence ratings ranged from 1–4 and 6–9, respectively, and they were also matched in word length, number of syllables and arousal levels (see Table 1). The task consisted of 24 practice trials (not scored) and 96 actual trials separated into four blocks of 24 trials. The first two trials of every block were not scored, leaving a total of 88 relevant trials for analysis. In each trial, participants viewed a single affective word presented centrally for 500ms followed by a 2500ms intertrial interval. Participants were instructed to indicate whether the valence of the current word (i.e., newer, incoming stimulus) had the same (match) or different (non-match) valence as the word two trials back by pressing the ‘1’ or ‘2’ key, respectively. There were 44 match trials (22 trials were positive-valenced stimuli, i.e., the current stimulus and the stimulus two trials back were both positive) and 44 non-match trials (21 trials were positive-valenced stimuli; i.e., the current stimulus was positive, but the stimulus two trials back was negative). To measure participants’ ability to update emotional information, we calculated the mean accuracy scores across all trials. “No responses” or omissions were counted as errors.

**Table 1:** Descriptives of the Emotional *n*-back word list.

		Frequency	
		Negative	Positive
Word Length (number of characters)			
	5–6	0	1
	7–8	32	30
	9–10	17	16
Number of syllables			
	1	0	1
	2	18	18
	3	26	22
	4	5	6
Arousal Level			
	2–3	1	0
	3–4	2	4
	4–5	17	15
	5–6	16	16
	6–7	11	10
	7–8	1	2
	8–9	1	0

The primary outcome measure for this version of the emotional *n-*back task is the accuracy scores [18] rather than RTs because of the difficulty of this task relative to the classical *n-*back or the emotional 2-back by Levens and Gotlib [13]. In the classical *n*-back, participants had to match the current stimulus with the stimulus *n* trials before. The emotional *2*-back that Levens and Gotlib [13] created is more similar to the classical *n*-back, in which participants had to simply match emotional faces. In this variant of the emotional *n*-back, there was an added complexity to the task: Participants had to first identify the valence of the new word before matching its valence with the word 2 trials back. This way, we ensure that participants were processing the emotional stimulus presented, and not just its perceptual features.

In addition, studies using response time (RT) scores have mean accuracy scores above 80% (e.g., [12]–[13]), making RT scores a reliable measure of ability. However, in the current task, participants tend to have lower mean accuracy scores (see Tables 2 and 3), making us hesitant to rely on RT scores as a measure of ability. However, for completeness, we also report RT results. For the RT scores, data preparation procedure can be found in [18], with the exception that the data used was the overall emotional *n*-back score for the current article, while data from specific valence conditions was used for the former article.

**Table 2:** Descriptive Statistics and Relationships of Different Variables in Study 1.

				Correlations				
		*M*	*SD*	1	2	3	4	
1.	Emotional *n*-back	.59	.13	–				
2.	Reappraisal	4.49	.97	.06	–			
3.	Rumination	1.89	.50	.06	.13	–		
4.	NA	2.13	.62	–.04	–.10	**.42**	–	
5.	PA	3.24	.70	–.05	**.26**	**−.21**	**−.20**	

*Note:* Pearson *r* correlations were used for Study 1. Emotional *n*-back performance is measured using accuracy scores. NA = Negative Affect; PA = Positive Affect. Significant correlations are indicated in bold (*p*<.05; 2-tailed).

**Table 3:** Descriptive Statistics and Relationships of Different Variables in Study 2.

				Correlations								
		*M*	*SD*	1	2	3	4	5	6	7	8	
Trait variables												
1.	Emotional *n*-back	.67	.14	–								
2.	Negative interference	17.53	104.38	.04	–							
Daily life variables												
3.	Reappraisal	18.27	19.07	**−2.92**	−.31	−						
						**.26/**						
4.	Rumination	27.29	26.07	−.68	−.27	**.14**	**–**					
						**.09/**	**.20/**					
5.	Anger/anxiety	13.90	14.42	−1.56	.49	**.17**	**.67**	**–**				
						**.11/**	**.29/**	**.59/**				
6.	Sadness/dysphoria	17.52	19.20	−.86	.71	**.15**	**.75**	**.45**	**–**			
						.01/	**−.31/**	**−.66/**	**−.71/**			
7.	Happiness	56.09	24.23	1.63	**−**1.79	.02	**−.34**	**−.20**	**−.31**	**−**		
						.01/	**−.28/**	**−.75/**	**−.55/**	**.59/**		
8.	Relaxation	58.16	24.66	1.39	**−**1.42	.01	**−.25**	**−.19**	**−.20**	**.48**	**−**	
						**.39/**	**.33/**	**.31/**	**.34/**	**−.07/**	**−.06/**	
9.	ER mean	23.92	14.79	**−**1.61	**−**.31	**.75**	**1.15**	**.32**	**.45**	**−.25**	**−.24**	

*Note:* For Study 2, since there is no straightforward way of calculating correlations in multilevel analyses the estimates provided for each pair of daily life variables are unstandardized coefficients from Level 1 multilevel models with one of the variables as an outcome measure and the other as a predictor (group mean-centered). As the multilevel estimates for the Level 1 variables are not symmetrical, the coefficients before the diagonal represent the coefficients obtained, in which the preceding variable was entered as the predictor and the succeeding variable as the outcome (e.g., reappraisal → rumination), while the coefficients after the diagonal represent the estimates, in which the succeeding variable was the predictor and the preceding variable was the outcome (e.g., rumination → reappraisal). The estimates provided for the relationship between a trait and daily variable (e.g., emotional n-back and reappraisal) are unstandardized coefficients with the daily variable as the Level 1 outcome and the trait variable as a Level 2 predictor.

Emotional *n*-back performance is measured using accuracy scores. Negative interference is measured using RTs, ER Mean = average emotion regulation use.

Significant correlations are indicated in bold (*p*<.05; 2-tailed).

#### Positive and negative affectivity

To measure positive (PA) and negative affectivity (NA), participants responded to the twenty-item Positive and Negative Affect Schedule (PANAS; [20]). Participants rated how much they experienced each of the 10 positive and 10 negative feelings in the past week using a scale from 1 (*very slightly or not at all*) to 5 (*extremely*) in the past week. PA and NA were calculated as the average score of the 10 positive and 10 negative feeling items, respectively.

#### Reappraisal

Participants completed the Emotion Regulation Questionnaire (ERQ; [9]). Participants rated from a scale of 1 (*strongly disagree*) to 7 (*strongly agree*) how much they typically use suppression (four items) and reappraisal (six items). Use of reappraisal was calculated as the average of the six reappraisal items in the questionnaire.

#### Rumination

Participants completed the 22-item Ruminative Response Style Questionnaire (RRS; [21]; Dutch version: [22]). They rated from a scale of 1 (*almost never*) to 4 (*almost always*) how often they ruminate when they are feeling sad or depressed. Habitual use of rumination was calculated as the average of all items in the questionnaire.

### Statistical Model

We ran a series of regression models to test whether the ability to update emotional information would moderate the relationship between reappraisal and rumination on one hand, and PA and NA, on the other hand. In the model, PA and NA were entered as dependent variables separately with overall emotional *n*-back and reappraisal scores (which were standardized), and their interaction as predictors. We also ran similar analyses, but with rumination (instead of reappraisal) as a predictor.

### Results

#### Preliminary Analyses

Descriptive statistics and intercorrelations are presented in Table 2. PA and NA were negatively correlated. Trait rumination was positively correlated with NA, and negatively correlated with PA. Trait reappraisal was positively correlated with PA, but did not have a significant relationship with NA. Moreover, we did not find a significant correlation between performance on the emotional *n*-back (accuracy scores) and any of the trait questionnaire measures.

There was also a positive correlation between the RT and the accuracy scores of the emotional *n*-back (*r* = .16, *p* = .02), implying a speed accuracy trade off. None of the trait measures correlated significantly with the RT scores of the emotional *n-*back (reappraisal: *r* = .03, *p* = .68; rumination: *r* = −.06, *p* = .39; PA: *r* = −.03, *p* = .63; NA: *r* = −.01, *p* = .83).

#### Reappraisal and NA

No significant main effects between reappraisal and NA (*β* = −.08, *SE* = .04, *p* = .07), and performance on the emotional *n*-back and NA (*β* = −.01, *SE* = .04, *p* = .85) were found. However, there was a significant interaction between reappraisal and performance on the emotional *n-*back (*β* = −.09, *SE* = .04, *p* = .02).

**Figure 1:**
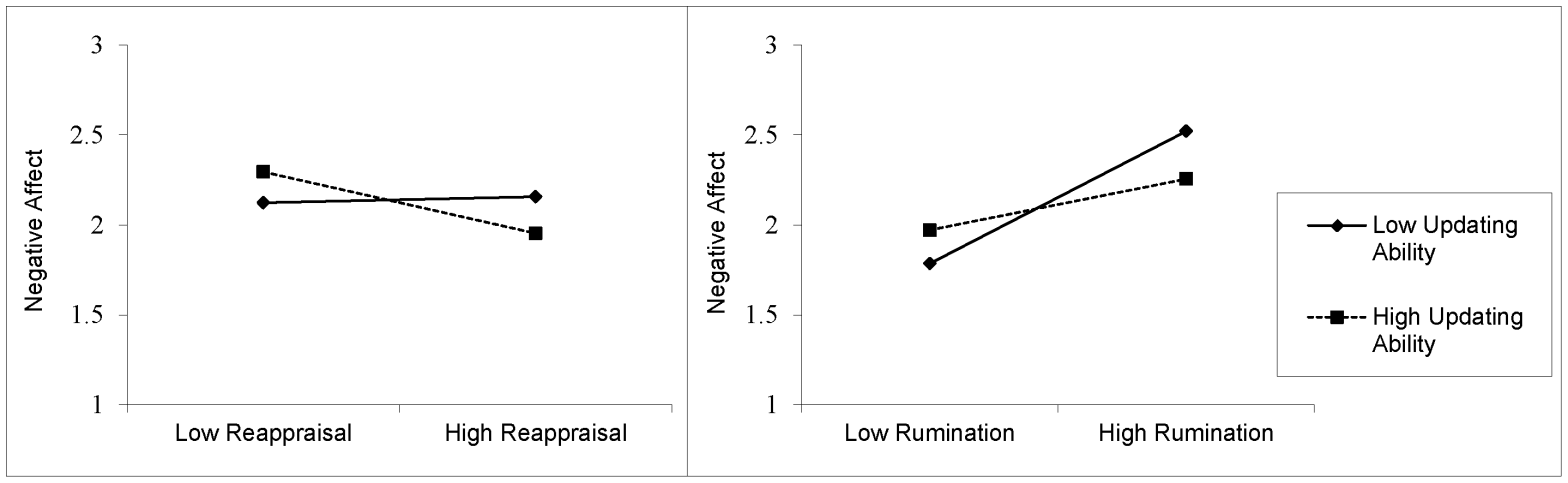
Illustration of the moderating effect of overall updating ability (as measured by the emotional *n*-back) on the relationship between reappraisal (left panel) and rumination (right panel) on NA at the trait level. Dashed and straight lines represent participants with low (-1 *SD*) and high (+1 *SD*) *n*-back scores, respectively. Low and high reappraisal scores are -1 *SD* and +1 *SD* from the overall mean, respectively. Low and high rumination scores were coded similarly.

We applied the simple slope analysis proposed by Preacher, Curran, and Bauer [23] to disentangle the interaction effect. Results revealed that participants with high updating ability (i.e., 1 *SD* above the mean) had a significant negative relationship between reappraisal and NA (*β* = −.17, *SE* = .07, *p* = .01). That is, for participants with high updating ability, habitually using reappraisal as an emotion regulation strategy was related to lower NA. However, for participants with low updating ability (i.e., 1 *SD* below the mean), the relationship between reappraisal and NA was non-significant (*β* = .02, *SE* = .06, *p* = .77). That is, the use of reappraisal was not associated with NA for participants with low updating ability. Figure 1 (left panel) illustrates this interaction effect.

For RT scores of the emotional *n*-back, no significant main effects between reappraisal and NA (*β* = −.07, *SE* = .04, *p* = .12), between emotional *n*-back and NA (*β* = −.01, *SE* = .04, *p* = .84) or interaction (*β* = −.04, *SE* = .04, *p* = .30) were found.

#### Reappraisal and PA

There was a significant main effect between reappraisal and PA (*β* = .19, *SE* = .05, *p*<.01) but not between reappraisal and the emotional *n*-back (*β* = −.05, *SE* = .05, *p* = .25). That is, participants who habitually used reappraisal as an emotion regulation strategy also experienced higher PA. No significant interaction was found (*β* = .04, *SE* = .04, *p* = .38).

For RT scores, there was a significant main effect of reappraisal on PA (*β* = .19, *SE* = .05, *p*<.01), but not between emotional *n-*back and PA (*β* = −.03, *SE* = .05, *p* = .56). No significant interactions were also found (*β* = .06, *SE* = .04, *p* = .17).

#### Rumination and NA

There was a significant main effect of rumination on NA (*β* = .26, *SE* = .04, *p*<.01), but not for the emotional *n*-back on NA (*β* = −.02, *SE* = .04, *p* = .59). However, a significant interaction between rumination and the emotional *n-*back task was found (*β* = −.11, *SE* = .04, *p*<.01).

Simple slope analysis [23] revealed that participants with high updating ability had a smaller positive slope (*β* = .14, *SE* = .05, *p*<.01) compared to those with low updating ability (*β* = .37, *SE* = .05, *p*<.01 ). This implies that although the habitual use of rumination implied higher NA scores, updating ability mitigated this effect. Indeed, participants with high (vs. low) updating ability and who habitually used rumination as an emotion regulation strategy reported less elevated NA. Figure 1 (right panel) illustrates this interaction effect.

For RT scores, there was a significant main effect of rumination on NA (*β* = .25, *SE* = .04, *p*<.01), and the interaction between rumination and the RT scores of the emotional *n*-back was marginally significant (*β* = −.07, *SE* = .04, *p* = .08). Main effect between emotional *n*-back and NA was non-significant (*β* = .00, *SE* = .04, *p* = .97).

#### Rumination and PA

There was a significant main effect of rumination on PA (*β* = −.15, *SE* = .05, *p*<.01), but not of the emotional *n*-back on PA (*β* = −.03, *SE* = .05, *p* = .56). That is, participants who habitually ruminated experienced a lower PA. No significant interaction was found (*β* = −.02, *SE* = .04, *p* = .71).

For RT scores, there was a significant main effect of rumination on PA (*β* = −.15, *SE* = .05, *p*<.01), but not between the emotional *n*-back and PA (*β* = −.03, *SE* = .05, *p* = .50). The interaction between rumination and emotional *n*-back was also non-significant (*β* = .00, *SE* = .05, *p* = .99).

#### Updating ability while controlling for effort or compliance

To discount the possibility that updating ability (as measured by accuracy scores) may be partly confounded by effort or compliance in doing the task, we also performed analyses only with participants who responded to at least 75% of the relevant trials (i.e., 66 out of 88 trials; *n* = 188). Results remained largely similar as presented above (with the exception that the interaction between reappraisal and emotional n-back became non-significant (*β* = −.06, *SE* = .04, *p* = .16). However, when applying the simple slope analysis, we found that participants with a high updating ability (1*SD* above the mean) still had a significant negative slope (*β* = −.16, *SE* = .07, *p* = .02), while the slope for participants with a low overall n-back (1*SD* below the mean) remained non-significant (*β* = −.04, *SE* = .06, *p* = .47).

### Discussion

The results of Study 1 showed that updating of emotional information was associated with the efficacy of reappraisal, supporting the findings of Schmeichel and colleagues [12]. Specifically, we found that updating moderated the relationship between reappraisal and NA, but not with PA. That is, the habitual use of reappraisal was associated with more decreased NA among participants with high, but not with low updating ability. Results also showed that updating moderated the relationship between rumination and NA, but not with PA. That is, the habitual use of rumination was associated with less elevated NA among participants with high (vs. low) updating ability.

Although the results of Study 1 give us important insight regarding the role of updating in regulating negative (and not of positive) emotions, it is still limited in three important ways: First, the use of trait questionnaires does not directly measure the use of emotion regulation strategies and its association with the intensity of emotions as they are experienced in everyday life. Second, it remains unclear whether the results are specific to updating ability, or are due to a shared ability with other executive processes that also moderate this relationship [24]. For correct conclusions to be drawn, it is important to exactly pinpoint the processes involved in our findings. Third, the range of emotions may be limited. The measure used to assess positive and negative emotions in this study (i.e., PANAS) only measures high arousal positive and negative emotions [25], which prevents us from making any conclusion about their low arousal counterparts.

## Study 2: Daily Life

Study 2 had three primary objectives. First, we aimed to examine whether the moderating effect of updating on the relationship between reappraisal and rumination on one hand, and affective experience on the other hand, would also occur in everyday life. In a previous study [7], we found that inhibition (specifically of negative information), an executive process that is related to updating [24], played a moderating role on the relationship between emotion regulation and emotional experience in everyday life. In this study, we wanted to examine whether updating would also moderate this relationship.

If updating would also moderate the relationship between rumination and reappraisal and affective experience, then it also becomes important to disentangle whether it is the shared relationship between updating and inhibition of negative information, or specific updating process that would moderate this relationship. To achieve this, we re-analyzed our experience sampling data set [7], [18], in which participants reported their emotions and their use of reappraisal and rumination 10 times a day, among others, over the course of one week, but this time we used the emotional *n*-back as the moderating variable. We also performed analyses with both inhibition (specifically of negative information as reported in [7]) and updating entered into the model to evaluate their unique contribution.

Finally, we wanted to explore whether the findings from Study 1 also apply to low arousal emotions. To test this hypothesis, we studied both high and low arousal positive and negative emotional states by including both high (happy) and low arousal positive items (relaxed); and both high (anger/anxiety) and low arousal negative items (sadness/dysphoria).

### Ethics statement

The study was conducted between February 2011 and May 2011. Ethical approval was requested since part of the study involved the use of physiological measures. This study was approved by the local ethics committee of the Faculty of Psychology and Educational Sciences, KU Leuven, Belgium. Written informed consent was obtained from all participants.

### Participants

Out of 439 first year undergraduates from University of Leuven who completed the Center for Epidemiologic Studies Depression Scale (CES-D; [26]), 100 participants, representing a wide, balanced, and uniform range of depression scores (range = 0–50, *M* = 19.27, *SD* = 12.53), were selected to take part in an experience sampling study. One participant withdrew early and four participants were excluded from data analyses (due to equipment malfunction, *n* = 3, or poor compliance, *n* = 1) leaving a final sample of 95 participants.

### Procedure

Participants received a Tungsten E2 palmtop computer along with instructions for general use and how to respond to the questions at each beep. For one week, participants carried the palmtop computer as they went about their daily activities. Palmtops were individually programmed (using ESP 4; [27]) to beep 10 times a day (during a 12-hour period) for seven consecutive days. On each day, the 12-hour sampling period was divided into 10 equal time blocks and one beep was programmed to occur randomly within each block. Items were presented in random order at each beep. Overall, participants responded to 91.5% (*SD* = 6.2) of the programmed beeps, demonstrating very good compliance.

Within the week of the experience sampling study, participants were required to attend a laboratory session to do additional cognitive tasks (i.e., emotional flanker [28], affective interference resolution task [7], emotional stroop-switching task [29] and the emotional *n*-back [18], which were administered in individual cubicles.

Since our previous findings have shown that the affective interference resolution task (specifically for negative stimuli) also moderated the regulation of emotional experience [7], we added this inhibition measure to our statistical models as a control variable. This will help ascertain that it is updating of emotional information, and not the shared relationship between inhibition of negative stimuli and updating that would explain the present findings. Descriptive statistics are presented in Table 3.

### Measures

#### Repeated assessment of emotions

At each sampling moment, participants reported how much they were feeling a number of specific emotions. Using a continuous slider scale that ranged from 1 (*not at all*) to 100 (*very much*), participants recorded their current levels of anger, sadness, dysphoria, anxiety, happiness and relaxation. A composite score for high and low arousal negative emotions was created by taking the mean score of the anger/anxiety and sadness/dysphoria items, respectively for each beep. High and low arousal positive emotions were taken from the items happiness and relaxation, respectively.

#### Repeated assessment of rumination and reappraisal

At each sampling moment, participants were asked to report on their use of a number of emotion regulation strategies. Of interest to the current study, an item on reappraisal (“Have you viewed the cause of your feelings from a different perspective since the last beep?”) and an item on rumination (“Have you ruminated since the last beep?”) were assessed. We have to note that the Dutch word used for “ruminated” was *piekeren*. “Piekeren” is the term that is used in everyday language to refer to rumination (and worrying). *Piekeren*, thus, broadly refers to recurrent negative thinking.

Participants also responded to items probing about other emotion regulation strategies (i.e., suppression, reflection, social sharing and distraction). Participants responded to these questions using a continuous slider that ranged from 1 (*not at all)* to 100 (*very much*). We also calculated a composite score for overall use of emotion regulation (ER mean) by taking the mean score of all emotion regulation items for each participant at each beep to represent how much participants tried to regulate their feelings in general at each moment (see also [7]).

#### Emotional n-back

Detailed description of the emotional *n-*back can be found in Study 1. Mean accuracy score was used as the main index of participant’s ability to update emotional information. Descriptive statistics are presented in Table 3.

#### Affective interference resolution task

We used the affective interference resolution task as a measure of inhibition. This task allows us to measure the level of interference from previously relevant (but is now irrelevant) emotional information in working memory. High levels of interference indicate difficulties inhibiting previously relevant information from accessing working memory. Detailed description of the task can be found in [7].

The task was composed of one practice block comprising eight trials (not scored) and 144 actual trials separated into eight blocks. Each trial began with a target set of four words presented around a fixation cross on the center of the screen for 1200ms, which was then followed by a delay of 3000ms, where only the fixation cross was presented. The fixation cross was then replaced by a probe word for 1500ms. Participants had to respond as quickly and accurately as possible whether or not the probe word was part of the current target set. Using the computer keyboard, participants pressed “1” if the answer was “Yes” and “2” if the answer was “No.”

Each target set contained two words that had been presented in the previous target set and two words that had not appeared in each of the two previous target sets. In addition, there was always at least one positive, one neutral and one negative word in each target set. An item never appeared in more than two consecutive trials.

There were four trial types (recent no, 45 trials; non-recent no, 36 trials; recent yes, 27 trials; and non-recent yes, 36 trials) which were divided equally among three valence conditions (*negative, neutral and positive*). A recent no response trial is a trial, in which the probe word did not match any items in the current target set (requiring a “no” response), but matched one of the words from each of the two previous target sets; a non-recent no response trial is a trial in which the probe word did not match any items in the current target set (requiring a “no” response) nor from the two previous target sets; a recent yes response trial is trial, in which the probe word matched an item in the current target set (requiring a “yes” response) and from the previous target set; and a non-recent yes response trial is a trial, in which the probe word matched an item in the current target set (requiring a “yes” response), but did not match any of the items from the previous set. The valence of each trial was determined by the probe word.

The difference score in accurate response times (RTs) between the recent no and non-recent no trials reflects the amount of interference that needs to be resolved [30]. Data preparation for the RT measures can be found in [7]. Interference scores were calculated separately for each valence (negative, neutral, positive). A higher score implies a higher amount of interference that participants needed to resolve [30]. Since results from our previous article [7] were focused on negative interference levels (calculated as the difference score between the interference scores from negative and neutral trials), this was also the variable used in this study. Descriptive statistics are presented in Table 3.

### Statistical model

To examine the impact of reappraisal and rumination on negative emotions in daily life, we used a similar multilevel regression modeling approach as presented in [7]. At Level 1, we modeled how affect (e.g., anger/anxiety) at time *t+1* can be predicted by the use of an emotion regulation strategy at time *t+1* (e.g., reappraisal, which was assessed “since the last beep”), controlling for the affect at the previous time *t*. This reflects the extent to which use of the emotion regulation strategy between times *t* and *t+1* is associated with a *change* in affect from time *t* to time *t+1*, where time *t* to *t+*1 refers to two consecutive beeps within the same day:
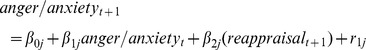



At Level 2, we modeled how the reappraisal-angry/anxious slope (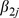) is a function of individual differences in updating ability:
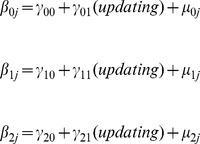



Such a multilevel model was estimated separately for each negative and positive emotion score (anger/anxiety, sadness/dysphoria, happiness, relaxation) and emotion regulation strategy (reappraisal/rumination).

### Results

#### Preliminary analyses

We present the descriptive statistics and associations among the different variables in Table 3. Rumination, reappraisal, and ER mean were all positively associated with the negative emotion measures (i.e., anger/anxiety, sadness/dysphoria) and negatively related with the positive emotion measures (except for reappraisal, which was non-significant). With regards to the relationships between the emotional *n-*back measure and daily life variables, there was a significant negative relationship between performance on the emotional *n*-back (accuracy scores) and average use of reappraisal. No other significant associations were found.

There was a positive correlation between the accuracy and RT scores of the emotional *n-*back (*r* = .23, *p* = .02), implying a speed-accuracy tradeoff. Using multilevel analyses, with daily variables as the Level 1 outcome and RT scores of the emotional *n-*back as Level 2 predictors, we found no significant associations between the RT scores of the emotional *n*-back and the daily life variables (rumination: *γ*_21_ = .21, *SE* = 1.54, *p* = .89; reappraisal: *γ*_21_ = −1.46, *SE* = 1.14, *p* = .20; anger/anxiety: *γ*_21_ = −1.59, *SE* = 1.17, *p* = .18; sadness/dysphoria: *γ*_21_ = −1.21, *SE* = 1.39, *p* = .39; happiness: *γ*_21_ = .98, *SE* = 1.19, *p* = .41; relaxation: *γ*_21_ = .96, *SE* = 1.11, *p* = .39; ER mean: *γ*_21_ = −.81, *SE* = 1.06, *p* = .45).

#### Reappraisal and negative emotions (uncorrected raw scores)

Results are presented in Table 4. We found a significant positive relationship between reappraisal and anger/anxiety, and between reappraisal and sadness/dysphoria (see γ_20_ scores in Table 4). This suggests that on average levels of updating ability, more use of reappraisal was associated with an increase of both high and low arousal negative emotions.

**Table 4:** Multilevel Analyses on the Moderating Effect of the Emotional n-back on the Relationship between Rumination/Reappraisal and Emotions in Daily Life.

				Anger/anxiety			Sadness/dysphoria			Happiness			Relaxation			
				Coef	SE	*p*	Coef	SE	*p*	Coef	SE	*p*	Coef	SE	*p*	
*Uncorrected raw scores*																
Rumination (β_2j_)																
		Intercept (γ_20_)		0.18	0.02	<.01	0.26	0.02	<.01	−0.27	0.02	<.01	−0.26	0.02	<.01	
		Slope (γ_21_)		−0.02	0.01	.08	−0.01	0.02	.61	−0.01	0.02	.73	−0.01	0.03	.63	
Reappraisal (β_2j_)																
		Intercept (γ_20_)		0.08	0.01	<.01	0.07	0.02	<.01	0.03	0.02	.12	0.03	0.02	.29	
		Slope (γ_21_)		−0.04	0.01	<.01	−0.01	0.02	0.38	-.00	0.02	.85	0.02	0.02	.23	
															
*Controlling for ER mean*																
Rumination (β_2j_)																
		Intercept (γ_20_)		0.13	0.02	<.01	0.21	0.02	<.01	−0.33	0.02	<.01	−0.29	0.03	<.01	
		Slope (γ_21_)		−0.02	0.01	.05	−0.01	0.02	.53	−0.01	0.02	.72	−0.01	0.02	.61	
Reappraisal (β_2j_)																
		Intercept (γ_20_)		−0.05	0.02	<.01	−0.09	0.02	<.01	0.14	0.02	<.01	0.13	0.02	<.01	
		Slope (γ_21_)		−0.03	0.01	<.01	−0.01	0.01	0.38	−.00	0.02	.86	0.02	0.02	.25	

*Note.* Coef = coefficient. In each analysis, the predictors at Level 1 were group-mean centered. Each of the Level 2 variables were grand-mean centered. The intercept (*γ* *_20_*) reflects the efficacy of an emotion regulation strategy on change in affect at average levels of updating ability across participants. The slope (*γ_21_*) reflects the association between updating ability and the efficacy of an emotion regulation strategy on change in affect.

Only the relationship between reappraisal and anger/anxiety (and not sadness/dysphoria) was moderated by performance on the emotional *n*-back (see γ_21_ scores in Table 4). Using simple slope analysis [23], we found that participants with high updating scores (i.e., 1 *SD* above the mean) experienced a smaller increase in high arousal negative emotions when reappraising (γ_21_ = .04, *SE* = .02, *p* = .01) compared to participants with low updating scores (i.e., 1 *SD* below the mean; γ_21_ = .11, *SE* = .02, *p*<.01).

RT scores of the emotional *n-*back did not moderate the relationships between reappraisal on one hand, and anger/anxiety (*γ*_21_ = −.03, *SE* = .02, *p* = .11) and sadness/dysphoria (*γ*_21_ = −.01, *SE* = .02, *p* = .77), on the other hand.

#### Rumination and negative emotions (uncorrected raw scores)

We found a significant positive relationship between rumination and anger/anxiety, and rumination and sadness/dysphoria (see γ_20_ scores in Table 4). That is, at average levels of updating ability, more use of rumination was associated with an increase of both high and low arousal negative emotions. Similar to the reappraisal results, only the relationship between rumination and anger/anxiety (and not sadness/dysphoria) was marginally significantly moderated by performance on the emotional *n*-back (see γ_21_ scores in Table 4). Using simple slope analysis [23], we found that participants with high updating ability had a smaller increase in high arousal negative emotions when ruminating (γ_21_ = .16, *SE* = .02, *p*<.01) compared to participants with low updating ability (γ_21_ = .21, *SE* = .02, *p*<.01).

RT scores from the emotional *n*-back moderated the relationship between rumination and anger/anxiety (*γ*_21_ = −.05, *SE* = .02, *p*<.01), but not with sadness/dysphoria (*γ*_21_ = −.02, *SE* = .02, *p* = .30).

#### Reappraisal and rumination and positive emotions (uncorrected raw scores)

To be consistent with the presentation of results in Study 1, we also report the moderating role of updating on the relationship of both reappraisal and rumination on change in positive emotions (see Table 4). At average levels of updating ability, rumination was related to a decrease of both high and low arousal positive emotions, while no significant relationship was found between reappraisal and positive emotions. Moreover, performance on the emotional *n*-back did not moderate the effect of both rumination and reappraisal on any of the positive emotion measures (see γ_21_ scores in Table 4).

RT scores of the emotional *n-*back did not moderate the relationships between reappraisal on one hand and happiness (*γ*_21_ = .01, *SE* = .02, *p* = .64) and relaxation (*γ*_21_ = .03, *SE* = .02, *p* = .15) on the other hand. It also did not moderate the relationships between rumination on one hand and happiness (*γ*_21_ = .03, *SE* = .02, *p* = .15) and relaxation on the other hand (*γ*_21_ = .03, *SE* = .03, *p* = .20).

#### Updating with rumination and reappraisal while controlling for ER mean

It was counterintuitive to find that reappraisal would be related to an increase in negative emotions. Similar to the argument we made in a previous article [7], this result could be explained by people’s tendency to just use various emotion regulation strategies when they are experiencing negative emotions. This assumption is supported in Table 3, where a positive correlation between overall use of emotion regulation strategies and the experience of negative emotions was found.

In order to control for this confound, we re-ran the same analyses while adding overall use of emotion regulation strategies as a Level 1 covariate. This allowed us to determine how much participants used rumination or reappraisal over and above their overall use of emotion regulation strategies since the last beep. Results are presented in Table 4.

#### Reappraisal and negative emotions (controlling for ER Mean)

After controlling for the overall use of emotion regulation strategies, we now found a significant negative relationship between reappraisal and anger/anxiety, and between reappraisal and sadness/dysphoria (see γ_20_ scores in Table 4). This suggests that on average levels of updating ability, relative high reappraisal was associated with a decrease of both high and low arousal negative emotions.

**Figure 2:**
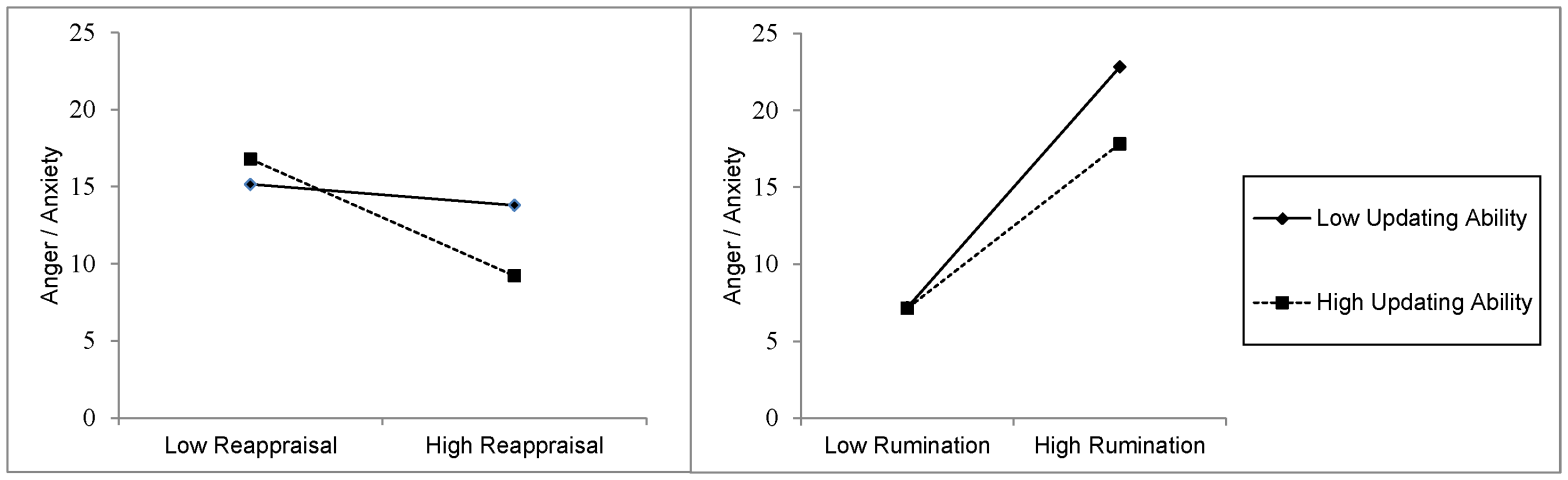
Illustration of the moderating effect of overall updating ability (as measured by the emotional n-back) on the relationship between reappraisal (left panel) and rumination (right panel) on anger/anxiety emotional experience (high arousal negative emotions) in daily life. Dashed and straight lines represent participants with low (-1 *SD*) and high (+1 *SD*) *n*-back scores, respectively. Low and high reappraisal scores are -1 *SD* and +1 *SD* from the overall mean, respectively. Low and high rumination scores were coded similarly.

In addition, we found that only the relationship between relative reappraisal and anger/anxiety was moderated by performance on the emotional *n*-back, while this was not the case for sadness/dysphoria (see γ_21_ scores in Table 4). Simple slope analysis [23] revealed that participants who were better at updating emotional stimuli on the *n*-back task experienced a larger decrease in high arousal negative emotions when they were reappraising more relative to employing other emotion regulation strategies (γ_21_ = −.08, *SE* = .02, *p*<.01). However, for participants with low updating ability, there was no change in high arousal negative emotions when they were using reappraisal more relative to other emotion regulation strategies (γ_21_ = −.01, *SE* = .02, *p* = .47). Figure 2 (left panel) illustrates this interaction effect.

RT scores from the emotional *n*-back moderated the relationship between reappraisal and anger/anxiety (*γ*_21_ = −.03, *SE* = .01, *p* = .03), but not with sadness/dysphoria (*γ*_21_ = −.01, *SE* = .02, *p* = .45).

#### Rumination and negative emotions (controlling for ER Mean)

After controlling for overall use of emotion regulation strategies, the results for rumination remained similar as the original analyses. Specifically, we found a significant positive relationship between relative rumination and anger/anxiety, and rumination and sadness/dysphoria (see γ_20_ scores in Table 4). In addition, only the relationship between rumination and anger/anxiety was moderated by performance on the emotional *n*-back, while this was not the case for sadness/dysphoria (see γ_21_ scores in Table 4). Simple slope analysis [23] revealed that participants who had high ability in updating emotional stimuli had a smaller increase in high arousal negative emotions when they were particularly ruminating relative to employing other emotion regulation strategies (γ_21_ = .11, *SE* = .02, *p*<.01) compared to those with lower updating ability (γ_21_ = .16, *SE* = .02, *p*<.01). See also Figure 2, right panel.

RT scores from the emotional *n*-back moderated the relationship between rumination and anger/anxiety (*γ*_21_ = −.06, *SE* = .02, *p*<.01), but not with sadness/dysphoria (*γ*_21_ = −.02, *SE* = .02, *p* = .18).

#### Reappraisal and rumination and positive emotions (controlling for ER Mean)

At average levels of updating ability, relative rumination was related to a decrease in positive emotions, while relative reappraisal was now related to an increase in positive emotions (see γ_20_ scores in Table 4). However, performance on the emotional *n*-back did not moderate the relationship between rumination and positive emotions, and reappraisal and positive emotions (see γ_21_ scores in Table 4).

RT scores from the emotional *n*-back moderated the relationship between reappraisal and relaxation (although marginal; *γ*_21_ = .04, *SE* = .02, *p* = .07), but not with happiness (*γ*_21_ = .02, *SE* = .02, *p* = .37). It also did not moderate the relationships between rumination on one hand and relaxation (*γ*_21_ = .03, *SE* = .02, *p* = .24) and happiness (*γ*_21_ = .02, *SE* = .02, *p* = .27) on the other hand.

#### Updating with rumination and reappraisal while controlling for ER mean

Similar to Study 1, to discount the possibility that updating ability (as measured by accuracy scores) may be partly confounded by effort or compliance in doing the task, we also performed analyses only with participants who responded to at least 75% of the relevant trials (i.e., 66 out of 88 trials; n = 92). Results remained the same.

#### Updating with rumination and reappraisal while controlling for inhibition of negative stimuli

To preclude the possibility that the shared variance of updating and inhibition of negative information, and not the specific updating of emotional information would predict the present findings, we ran the same models as above (i.e., models that include the ER Mean as a Level 1 predictor) while controlling for inhibition of negative information. That is, in addition to the updating measure, the negative interference level was also included as a predictor for the person-specific intercept and slope values at Level 2.

Results showed that after controlling for inhibition of negative information, updating of emotional information remained a significant moderator of the relationship between rumination and reappraisal on one hand, and high arousal negative emotions on the other hand (see Table 5). This suggests that it is unlikely that the shared relationship between these two measures could explain the moderating effect of updating on the efficacy of rumination and reappraisal on high arousal negative emotions.

**Table 5:** Multilevel Analyses on the Moderating Effect of the Emotional n-back on the Relationship between Rumination/Reappraisal and Emotions in Daily Life after Controlling for Inhibition of Negative Information.

				Anger/anxiety			Sadness/dysphoria			Happiness			Relaxation		
				Coef	SE	*p*	Coef	SE	*p*	Coef	SE	*p*	Coef	SE	*p*
Rumination (β_2j_)															
		Intercept (γ_20_)		0.13	0.02	<.01	0.22	0.02	<.01	−0.33	0.02	<.01	−0.29	0.03	<.01
		Slope (γ_21_)		−0.03	0.01	.04	−0.01	0.02	.48	−0.00	0.02	.81	−0.01	0.02	.62
		Slope (γ_22_)		0.03	0.01	.06	0.04	0.02	.02	−0.04	0.02	.02	−0.01	0.02	.57
Reappraisal (β_2j_)															
		Intercept (γ_20_)		−0.04	0.02	<.01	−0.09	0.02	<.01	0.14	0.02	<.01	0.12	0.02	<.01
		Slope (γ_21_)		−0.03	0.01	<.01	−0.01	0.01	.40	−0.01	0.02	.76	0.02	0.02	.27
		Slope (γ_22_)		0.01	0.01	.32	0.03	0.01	.03	−0.04	0.02	.01	−0.03	0.03	.31

*Note.* Coef = coefficient. In each analysis, the predictors at Level 1 were group-mean centered. Each of the Level 2 variables were grand-mean centered. The intercept (*γ_20_*) reflects the efficacy of an emotion regulation strategy on change in affect at average levels of updating ability across participants. The slope (*γ_21_*) reflects the association between updating ability and the efficacy of an emotion regulation strategy on change in affect. The slope (*γ_22_*) reflects the association between negative interference levels (from the affective interference resolution task) and the efficacy of an emotion regulation strategy on change in affect.

### Discussion

In Study 2, we aimed to examine whether updating of emotional information would moderate the relationship between reappraisal and rumination, on one hand, and affective experience, on the other hand, in everyday life, and whether this effect would remain even after controlling for inhibition of negative information. Moreover, we wanted to examine whether this effect would be found not only with high but also with low arousal positive and negative emotions.

Results revealed that updating of emotional information moderated the effect of rumination and reappraisal on high arousal negative emotions (i.e., anger/anxiety) alone. We did not find this for low arousal negative emotions (i.e., sadness/dysphoria) or with positive emotions (i.e., happiness and relaxation). Specifically, participants who were better at updating emotional information experienced a larger decrease and a smaller increase in high arousal negative emotions when they were particularly reappraising and ruminating, respectively, relative to using other emotion regulation strategies. These results remained even after controlling for the ability to inhibit negative information.

## General Discussion

The findings of the present study provided empirical support to the hypothesis put forward by Schmeichel and colleagues [12]: Individual differences in cognitive ability moderate the success or failure of emotion regulation strategies. Specifically, we found that updating of emotional information moderated the efficacy of both rumination and reappraisal on high arousal negative emotions at the trait level (Study 1) and in daily life (Study 2). That is, participants who were better at updating emotional information in WM, and habitually used rumination and reappraisal reported less elevated and more decreased high arousal negative emotions, respectively (Study 1). They also experienced a smaller increase and a larger decrease in high arousal negative emotions when ruminating and reappraising, respectively, in everyday life, and this result remained even after controlling for the moderating effect of negative interference levels (Study 2).

We did not expect that the relationship between updating ability and efficacy of rumination and reappraisal would consistently be limited to high arousal negative emotions. Although unforeseen, it coincides with previous work on the relationship between emotion and executive control [31]. Indeed, it has been hypothesized that particularly emotional content high in threat impair executive resources as attentional control is diverted towards the processing of high threat information (see [31]). Once executive resources are depleted (even temporarily), fewer resources can be recruited to do subsequent tasks that also require executive control, such as emotion regulation [32]. High threat situations are of course primarly implicated in the generation of high arousal negative emotions such as anger and anxiety. We propose that participants with high updating ability would have more executive resources to spare after processing high threat emotional information, and therefore would still have enough resources to effectively regulate these emotions. This would not be the case for participants with low updating ability. After processing high threat emotional information, they would probably have depleted most of their executive resources, leaving them with fewer resources to effectively regulate their emotional experience in response to such events.

We also reiterate here the rationale for using accuracy scores as our main dependent variable rather than RTs. The current variant of the emotional *n-*back task is more difficult than the classical *n-*back (e.g., [33]) or the emotional 2-back by Levens and Gotlib [13]. In both of these tasks, participants had to match the current stimulus with the stimulus *n* trials before. In our variant of the emotional *n*-back, there was an added complexity to the task: Participants had to first identify the valence of the new word before matching its valence with the word 2 trials back. This way, we ensure that participants were processing the emotional stimulus presented, and not just its perceptual features. However, because of this added complexity, accuracy scores on our variant of the emotional *n*-back were not as high (59% for Study 1; 67% for Study 2) compared to both the classical *n*-back (e.g., visual 2-back: 91%–92%; [33]) and emotional *2*-back by Levens and Gotlib (76%–94%; [13]) that are reported in the literature. The relatively lower accuracy scores for this variant of the *n*-back also make us hesitant to rely on RT scores, as RT scores only become a good measure of ability under the condition that accuracy levels are high (note that we nevertheless report RT results for completeness). Although we did not find any significant moderating effect of the RT scores in Study 1, we did in Study 2. In Study 2, we consistently found that longer RTs predicted smaller increase and larger decrease in high arousal negative emotions when ruminating and reappraising, respectively. However, this counterintuitive result can be explained by the positive correlation between RT and accuracy scores found in both studies. This implies a speed-accuracy trade-off, in which spending a longer time in responding results in better performance for this task [34].

Although it is more appropriate to use accuracy scores as a measure of performance on this variant of the emotional *n*-back [18], we cannot discount the possibility that this method of assessing updating ability may be partly confounded by effort or compliance in doing the task. Although this predicament is not uncommon when using cognitive ability measures (e.g., see [35]), it is possible to minimize this confound by removing participants who have a relatively large amount of “no responses” or omissions in the emotional *n-*back. When reanalyzing the data set with participants who responded to at least 75% of all relevant trials (i.e., 66 out of 88 trials), we found that our findings remained largely the same, suggesting that it seems unlikely that our results can be explained in terms of effort alone.

This study extends previous work in several ways. First, we defined and tested cognitive ability in a different way. We focused on updating (as measured by the emotional *n*-back), which is an executive process that requires modifying the contents of WM to accommodate incoming newer relevant information [11]. Previous work on the moderating effect of cognitive ability on successful emotion regulation primarily focused on general working memory capacity (WMC; [12], [36]), which is defined as the “ability to sustain goal-relevant information processing in the presence of alternative goals or other distractions” ([12]: p. 1527). Although these two processes are included in the broader construct of WM or executive functions, there is growing evidence that measures of WMC (e.g., operation span) and updating (e.g., *n*-back) do not load on the same WM component (e.g., [33]). However, when we combine findings from previous studies [12], [36] and the current investigation, the findings inform us that both WMC and updating play a moderating role on successful emotion regulation. Whether it is their shared variance or that they each independently predict successful emotion regulation remains an important question in the literature and should be investigated further in the future.

Second, to our knowledge, there are few, if any, studies that looked at the moderating role of cognitive ability on the success or failure of rumination (for exception, see [7]). Previous studies have primarily focused on emotion regulation strategies, such as reappraisal [12], [36] and suppression [12]. In the present study, we found that although rumination was associated with greater experience of high arousal negative emotions, better updating ability curbed this effect. That is, people who were better at updating tend to experience less elevated high arousal negative emotions at the trait level, and a smaller increase in high arousal negative emotions in daily life when they ruminated. This finding is important because it supports the notion that rumination involves elaboration of negative material that is already present in WM [13]. A person with poor updating ability would have difficulties allowing *newer* information to feed into WM, which would result into a continuous elaboration of current negative material in WM. A person with better updating ability would have a higher chance of breaking this cyclic process. As new information feeds into WM, the contents of WM also change, which then interrupts the elaboration of negative material when ruminating. As a result, this lessens the maladaptive effects of rumination.

Importantly, our results also showed that the role of updating on efficacy of emotion regulation extends to everyday life, going beyond results found in the laboratory. This methodological difference is important since results found in daily life give insight into experiences in real time, which is distinctive from trait questionnaires, wherein responses are reliant on how things are remembered [37]. Indeed, when participants were asked how much they regulated their emotions (since the last beep) and how they currently felt, results showed that participants who were better at updating showed a lesser increase in high arousal negative emotions when they ruminated, and a larger decrease in high arousal negative emotions when they reappraised. These findings were similar to what we found with trait questionnaires in Study 1, and what Schmeichel and colleagues [12] found in an experimental study (at least for reappraisal). The combination of various methodologies used to assess the moderating role of updating on efficacy of emotion regulation give stronger empirical support regarding the importance of cognitive ability in implementing emotion regulation strategies.

The current results give additional support to the assumption that updating ability is beneficial for overall well-being. Our previous findings have focused on the relationship of updating of positive information and the experience of subjective well-being or happiness [18]. The current findings point towards the importance of updating ability when regulating high arousal negative emotions. Specifically, the results seem to suggest that updating ability is a necessary component for the successful down-regulation of high arousal negative emotions when reappraising, and acts as a protective mechanism against the increase of high arousal negative emotions when ruminating.

Our results suggest ways to inform therapeutic interventions for mood disorders. While many therapeutic interventions are aimed at changing the strategies that people use to regulate their emotions (e.g., to reduce the use of rumination, or to increase the use of reappraisal), our findings indicate that training people’s updating ability could have the added benefit that it directly modulates the impact of both strategies in therapeutic ways.

However, one interesting area for future research is to investigate whether the relationship between updating and efficacy of rumination and reappraisal would differ between men and women. Because of the disproportionate number of men and women in our two studies, it was not possible to examine such gender differences in the present manuscript.

### Conclusion

In conclusion, the present study reveals that the ability to update emotional information plays a role in the efficacy of rumination and reappraisal on regulating high arousal negative emotions. In two studies, results showed that people with better updating ability tend to experience more decreased and less elevated high arousal negative emotions when reappraising and ruminating, respectively, compared to others. These findings indicate that updating ability may be a critical component for successful emotion regulation.
